# Clinical and Angiographic Profile in Non-ST Elevation Acute Coronary Syndrome (NSTE-ACS) and Chronic Stable Angina: A Tertiary Care Centre-Based Cohort Study From Southern Indian Population

**DOI:** 10.7759/cureus.38369

**Published:** 2023-05-01

**Authors:** Venkatesh Raju

**Affiliations:** 1 Cardiology, Thoothukudi Medical College, Thoothukudi, IND

**Keywords:** non-st elevated myocardial infarction, coronary artery disease, coronary angiography, unstable angina, acute coronary syndrome

## Abstract

Purpose

This study aimed to assess the clinico-demographic profile, risk factors, and pattern of coronary involvement in non-ST elevation acute coronary syndrome (NSTE-ACS) and chronic stable angina (CSA).

Methods

This was a retrospective study conducted in a tertiary care hospital catering mainly to the rural population in Southern India from January 2020 to July 2022. Data from 333 patients with NSTE-ACS and CSA were analyzed during the study period for the clinico-demographic profile, risk factors, and pattern of coronary involvement in angiography.

Results

The mean age at presentation was 56.05±9.31. Significant obstructive coronary artery disease was present in 234 (70.3%) patients. Overall, single, double, and triple vessel disease occurred in 26%, 20%, and 32.4% of patients with NSTE-ACS and chronic stable angina. Sixty percent of the diabetic and hypertensive patients (n=92/153) had multivessel involvement. Left main coronary artery disease was present in 40 patients (12%).

Conclusion

Unstable angina was the most common clinical presentation. Left anterior descending (LAD) was the most common coronary vessel to be involved with occurrence of triple vessel disease in 32% of the study population. Multivessel involvement was more common in the diabetic and hypertensive groups.

## Introduction

Ischemic heart disease may manifest as either chronic stable angina (CSA) or acute coronary syndrome (ACS). The spectrum of ACS includes ST elevation myocardial infarction (STEMI) and non-ST elevation ACS (NSTE-ACS) which comprises unstable angina and non-ST elevation MI (NSTEMI). The proportion of ACS attributed to NSTEMI continues to increase, while STEMI is declining. This study aimed to assess the coronary angiographic profile in NSTE-ACS and CSA in the Southern Indian population.

## Materials and methods

This was an observational study conducted at Thoothukudi Medical College and Hospital over a period of two years from April 2020 to July 2022. The tertiary care center caters to the rural population of Southern India in Tamil Nadu. The study was done as per the ethical standards set by the Institute Ethics Committee following the 1964 Helsinki Declaration and its amendments. There was a waiver of consent since it was a record-based study and the protocol for the study was approved by the institute ethics committee, Thoothukudi Medical College.

Records of 333 patients with NSTE-ACS and chronic stable angina admitted to Thoothukudi Medical College were analyzed. The patients were categorized as unstable angina, NSTEMI, and CSA. Valvular heart disease and cardiomyopathy were excluded from the study. Demographic details including age and gender were recorded in a predesigned clinical proforma. Any history of smoking, past history of coronary artery disease, and presence of comorbidities, such as diabetes and hypertension, were recorded. The angiographic details of the study population were entered.

Outcome measures

Coronary stenosis was classified as moderate if the narrowing was between 50% and 70% and as severe and significant when the diameter reduction was 70% or more in at least one of the major epicardial coronary arteries. Obstructive coronary artery disease (CAD) was also defined as at least 50% stenosis of the left main coronary artery (LMCA). Normal coronaries referred to the absence of any disease in the left anterior descending (LAD), left circumflex (LCX) and its branches, right coronary artery (RCA), and LMCA. Based on the involvement of the number of vessels, they were further classified as single vessel, double vessel, and triple vessel disease [1].

Statistical analysis

Data were analyzed using the software STATA 15.0 (Texas, USA: Stata Corp.). The normality of the continuous variables was tested and mean with standard deviation was used to present Gaussian variables and median with range is used to present non-Gaussian variables. The groups were compared using Fisher’s exact test or chi-square test for categorical variables and Student’s t-test for continuous variables.

## Results

Baseline characteristics

Data from 333 patients were analyzed in the study. There were 234 (70.3%) male and 99 (29.7%) female patients. The mean age of the study population was 56.05±9.31 years. Mean age at presentation in the male group was 55.43­±9.60 and in the female group was 57.41­±8.42. A total of 158 (47.4%) patients were diabetic, 153 (45.9%) were hypertensive, and 180 (54%) patients were smokers. Eighty patients (24%) had chronic stable angina; 211 (63.4%) had unstable angina (UA) and 42 (12.6%) patients had NSTEMI. Baseline characteristics are summarized in Tables 1, 2.

**Table 1:** Baseline characteristics of the study participants. NSTEMI: non-ST elevation myocardial infarction; CSA: chronic stable angina

Baseline characteristics	Baseline values
Average age of overall population (years) mean±SD	56.05±9.31
Average age of male (years) mean±SD	55.43­±9.60
Average age of female (years) mean±SD	57.41­±8.42
Unstable angina, n (%)	211 (63.4%)
NSTEMI, n (%)	42 (12%)
CSA, n (%)	80 (24%)
Diabetes mellitus, n (%)	158 (47.4%)
Hypertension, n (%)	153 (45.9%)
Smoking, n (%)	180 (54%)

**Table 2:** Patient characteristics in various subgroups. NSTEMI: non-ST elevation myocardial infarction; NSTE-ACS: non-ST elevation acute coronary syndrome; CSA: chronic stable angina

Variables	Mean age	Male, n (%)	Mean age in male	Female n (%)	Mean age in female	Diabetic n (%)	Hypertensive n (%)
Unstable angina (n=211)	55.66±9.56	151 (71.6)	54.95±9.83	60 (28.4)	57.43±8.67	100 (47.4)	97 (45.9)
NSTEMI (n=42)	56.57±9.15	29 (69.1)	56.03±9.43	13 (30.9)	58.31±8.51	18 (42.8)	16 (38.1)
NSTE-ACS (n=253)	56.11±9.35	180 (71.1)	55.49±9.63	73 (28.8)	57.87±8.59	118 (46.6)	113 (44.7)
CSA (n=80)	56.74±8.8	54 (67.5)	56.46±9.20	26 (32.5)	57.31±8.06	39 (48.7)	38 (47.5)

Outcome data

Significant obstructive CAD occurred in 234 (70.3%) patients. The angiographic profile of the study population is detailed in Tables 3-5.

**Table 3:** Angiographic profile of patients. CAD: coronary artery disease; NSTEMI: non-ST elevation myocardial infarction; NSTE-ACS: non-ST elevation acute coronary syndrome; CSA: chronic stable angina

Disease entity	Total number of patients	Normal coronaries, n (%)	Non-obstructive/minimal CAD n (%)	Obstructive CAD	
				Moderate (50 to <70%), n (%)	Significant (>70%), n (%)
Unstable angina	211	12 (5.7)	30 (14.2)	24 (11.4)	145 (68.7)
NSTEMI	42	0 (0)	2 (4.7)	7 (16.7)	33 (78.6)
NSTE-ACS	253	12 (4.7)	32 (12.6)	31 (12.2)	178 (70.3)
CSA	80	4 (5)	13 (16.2)	7 (8.7)	56 (70)
Total	333	16 (4.8)	45 (13.5)	38 (11.4)	234 (70.3)

**Table 4:** Coronary vessel involvement in NSTE-ACS and CSA. SVD: single vessel disease; DVD: double vessel disease; TVD: triple vessel disease; LMCA: left main coronary artery; LAD: left anterior descending artery; LCX: left circumflex artery; RCA: right coronary artery; NSTEMI: non-ST elevation myocardial infarction; NSTE-ACS: non-ST elevation acute coronary syndrome; CSA: chronic stable angina

Disease entity	Total number of patients	SVD	DVD	TVD	LMCA	LAD>70%	LCX>70%	RCA>70%
Unstable angina	211	57 (27%)	46 (21.8%)	54 (25.6%)	15 (7.1%)	106 (50.2)	81 (38.4)	83 (39.3)
NSTEMI	42	10 (24%)	5 (11.9%)	25 (59.5%)	10 (23.8%)	31 (73.8)	22 (52.4)	25 (59.5)
NSTE-ACS	253	67 (26.5)	51 (20.1)	79 (31.2)	25 (9.8)	137 (54.1)	103 (40.7)	108 (42.7)
CSA	80	20 (25%)	18 (22.5%)	29 (36.2%)	15 (18.7%)	47 (58.7)	38 (47.5)	35 (43.7)
Total	333	87 (26.1%)	69 (20.7)	108 (32.4%)	40 (12.01%)	184 (55.2)	141 (42.3)	143 (42.9)

**Table 5:** Specific coronary vessel involvement in NSTE-ACS and CSA. LAD: left anterior descending artery; LCX: left circumflex artery; RCA: right coronary artery; NSTEMI: non-ST elevation myocardial infarction; NSTE-ACS: non-ST elevation acute coronary syndrome; CSA: chronic stable angina

Disease entity	Only LAD, n (%)	Only RCA, n (%)	Only LCX, n (%)	LAD and LCX, n (%)	LAD and RCA, n (%)	LCX and RCA, n (%)
Unstable angina (211)	37 (17.5)	9 (4.2)	11 (5.2)	11 (5.2)	24 (11.4)	10 (4.7)
NSTEMI (42)	8 (19)	1 (2.3)	1 (2.3)	1 (2.3)	4 (9.5)	0 (0)
NSTE-ACS (253)	45 (17.7)	10 (3.9)	12 (4.7)	12 (4.7)	28 (11.1)	10 (3.9)
CSA (80)	16 (20)	2 (2.5)	2 (2.5)	10 (12.5)	6 (7.5)	2 (2.5)

Left anterior descending (LAD) artery was the most common coronary vessel to be involved (n=184) and LMCA was involved only in 40 patients. On analysis of the multivessel involvement group, 70% were male; 57% were diabetic; 50% were hypertensive and 56% presented with unstable angina (Table 6). NSTEMI group had significant obstructive CAD in 78.6% of patients with triple vessel involvement in 59.5%. Similarly, 92 patients (60%) in the diabetic and hypertensive group had multivessel involvement (double vessel disease {DVD} in 32 and triple vessel disease {TVD} in 53).

**Table 6:** Characteristics of patients with multivessel involvement. NSTEMI: non-ST elevation myocardial infarction; NSTE-ACS: non-ST elevation acute coronary syndrome; CSA: chronic stable angina

Total patients	n (%), total n= 177
Male	124 (70)
Mean age	58.02±9.04
Diabetes	102 (57.6)
Hypertensive	90 (50.8)
NSTEMI	29 (16.4)
Unstable angina	100 (56.5)
NSTE-ACS	129 (72.8)
CSA	47 (26.6)

## Discussion

Main findings with interpretation

The mean age at presentation of NSTE-ACS was 56.11±9.35 years with a much earlier age at presentation in the male population (55.49±9.63). The disease presentation in the Southern Indian population is much earlier when compared to Western literature where the average age ranges from 60 to 70 years [2-4]. Similarly, the mean age at presentation of chronic stable angina was 56.7±8.8 years. Whereas the Clinical Outcomes Utilizing Revascularization and Aggressive Drug Evaluation (COURAGE) trial showed the mean age at presentation to be 62±10 years [5]. The mean age at presentation as per the current study is 56 years which is similar to other Indian studies [6]. The identification of the background risk factors for such earlier presentation in Indian population will aid in devising apt preventive strategies.

Predominantly, the male gender was affected with the disease in a proportion of 71.1% and 67.5% in the NSTE- ACS and chronic stable angina groups, respectively. Third Randomized Intervention Trial of Unstable Angina (RITA-3) and The Treat Angina with Aggrastat and Determine Cost of Therapy with an Invasive or Conservative Strategy (TACTICS) Thrombolysis in Myocardial Infarction (TIMI)-18 (TACTICS-TIMI-18) trial also had similar male-affected study groups of 63% and 66%, respectively [7,8]. The COURAGE trial and International Study of Comparative Health Effectiveness With Medical and Invasive Approaches (ISCHEMIA) trial, respectively, report a presentation of CSA in 85% and 77.4% males, while our study in comparison reported CSA in 67.5% males [5,9]. The major associated factors include diabetes mellitus, hypertension, and smoking in the index study. The striking difference from the Western cohort is the increased prevalence of comorbidities, such as hypertension and diabetes mellitus, in Indian population. Invasive versus Conservative Treatment in Unstable Coronary Syndromes (ICTUS) trial results showed only 14% patients with diabetes mellitus in comparison to 47% patients in this study [10]. Further multivessel involvement in the diabetic group with earlier age of occurrence emphasizes the critical need for diabetes control and prevention measures to curtail the rising trend of CAD in India [11].

On analysis of the angiographic patterns of CAD in NSTE-ACS, 70% had significant obstructive CAD with LAD being the most common vessel to be involved (54.1%). This observation is consistent with other studies which show LAD (50%) as the most common culprit vessel [12,13]. Normal coronaries occurred in 4.8% with non-obstructive lesions in 13.5% patients. LMCA disease occurred in 9.8% patients of NSTE-ACS group which is similar to the Veterans Affairs Non-Q-Wave Infarction Strategies in Hospital (VANQWISH) trial and FRagmin and Fast Revascularization during Instability in Coronary Artery Disease (FRISC-II) trial wherein the reported involvement was 8% [2,3]. The NSTE-ACS patients had predominant multivessel involvement with 31.2% triple vessel disease and 20.1% double vessel disease. These findings are similar to the data from landmark trials which are summarized in Table 7.

**Table 7:** Comparative characteristics of patient cohort of NSTE-ACS in other studies. LAD: left anterior descending artery; SVD: single vessel disease; DVD: double vessel disease; TVD: triple vessel disease; CAD: coronary artery disease; RITA-3: Third Randomized Intervention Trial of Unstable Angina; VANQWISH: Veterans Affairs Non-Q-Wave Infarction Strategies in Hospital; FRISC-II: FRagmin and Fast Revascularization During Instability in Coronary Artery Disease; TIMI: Thrombolysis in Myocardial Infarction; NSTE-ACS: non-ST elevation acute coronary syndrome; LMCA: left main coronary artery; ISAR-COOL: Intracoronary Stenting with Antithrombotic Regimen Cooling-off trial

Variables	Current study	VANQWISH trial [2]	ISAR-COOL trial [4]	RITA-3 trial [8]	FRISC-II trial [3]	TIMI-IIIB trial [13]
Age (years) Mean	55	62	70	62	66	59
Male gender (%)	71.1	97	77	62	69.5	66
Hypertension (%)	44.7	54	86	34.9	30.2	42
Diabetes mellitus (%)	46.6	26	29	13.5	12.2	
Smoking (%)	51%	43	-	-	30	37
Minimal CAD (%)	12.6	3.6	11.2	-	14/9 (invasive/ non-invasive group)	15.6
SVD (%)	26.5	16.7	19.3	33	30/26 (invasive/non-invasive group)	37.4
DVD (%)	20.1	19.6	24.1	24	26/28 (invasive/ non-invasive group)	31.2
TVD (%)	31.2	33.4	45.3	22	23/30 (invasive/ non-invasive group)	16.3
LMCA involvement (%)	9.8	7.5	-	-	8/8	4.5

Chronic stable angina patients who were not responding to medical management were referred for coronary angiogram (CAG). Among them, 18.7% had left main obstructive disease and approximately 16.2% had no critical obstruction. Single, double, and triple vessel diseases were present in 26%, 20%, and 32% patients, respectively, in the index study. Similarly, the angiographic findings of ISCHEMIA trial revealed 22.5%, 33.7%, and 43.6% of single, double, and triple vessel diseases, respectively [9].

Strengths and limitations

The strength of the study is the in-depth analysis of the angiographic profile and risk characterization of NSTE-ACS. While most of the studies are from Western literature, the study fills the lacunae of a dearth of knowledge about the disease characteristics in the Southern Indian population. The limitations include the sample size and retrospective nature of the study. Future prospective studies and risk factor analysis with follow-up will aid in the risk categorization, prognostication, and targeted preventive measures in the identified high-risk population.

## Conclusions

The presentation of NSTE-ACS occurs a decade earlier in the Southern Indian population when compared to the West with a higher proportion of non-communicable diseases, such as diabetes and hypertension. Multivessel involvement was more commonly observed in males, diabetic, hypertensive, and unstable angina patients. The distribution of coronary angiographic profile of the study population is nearly similar to the Western cohort.
